# Corporeal Compression at the Onset of Septic shock (COCOONs): a compression method to reduce fluid balance of septic shock patients

**DOI:** 10.1038/s41598-019-47939-2

**Published:** 2019-08-09

**Authors:** Auguste Dargent, Audrey Large, Agnès Soudry-Faure, Jean-Marc Doise, Caroline Abdulmalak, Lysiane Jonval, Pascal Andreu, Jean-Baptiste Roudaut, Sébastien Prin, Pierre-Emmanuel Charles, Didier Payen, Jean-Pierre Quenot, The COCOONs study group, Maël Hamet, Thomas Poussant, Martial Delorme, Adrien Lhoumeau, Thierry Comte, Abderrahmane Bourredjem

**Affiliations:** 1 Department of intensive care, François Mitterrand University Hospital, Dijon, France; 2 CHU Dijon Bourgogne, Clinical Research Unit-Methodological Support Network, Dijon, France; 3 Department of intensive care, William Morey Hospital, Chalon-sur-Saône, France; 4 INSERM Research Center UMR 1231 and LabEx LipSTIC, Lipness Team, University of Burgundy, Dijon, France; 5 UMR 1160 INSERM, Paris 7 University, AP-HP, Paris, France; 6 INSERM CIC 1432, Clinical Epidemiology, University of Burgundy, Dijon, France

**Keywords:** Therapeutics, Therapeutics, Phase II trials, Phase II trials

## Abstract

Fluid overload in septic intensive care unit (ICU) patients is common and strongly associated with poor outcome. There is currently no treatment for capillary leak, which is mainly responsible for high positive fluid balance (FB) in sepsis. We hypothesized that increasing interstitial pressure with extensive corporeal compression would reduce FB. The objective of this study was to evaluate the feasibility, efficacy, and safety of a compression treatment during sepsis. This pilot, two-center, single-arm trial enrolled critically ill, non-surgical, septic patients receiving mechanical ventilation. The therapeutic intervention was the early application of compression bandages on more than 80% of the body surface. The primary outcome was negative net FB on day 7. The primary endpoint was reached in 29 of 45 patients (64%) with available data, for a planned objective of 26. By day 4, cumulative FB was 7280 ml [3300–9700]. SOFA- and aged-matched patients from a historical cohort had a significantly higher FB at 1, 2 and 7 days. Tolerance was good, although low-stage pressure ulcers were observed in 16 patients (26%). No effect on intra-abdominal pressure or respiratory plateau pressure was observed. In conclusion, corporeal compression demonstrated potential efficacy in limiting FB during septic shock, with acceptable feasibility and tolerance.

## Introduction

Fluid resuscitation is a cornerstone of supportive therapy at the acute phase of septic shock^1^ but it is associated with the risk of fluid overload. Due to the inflammation-induced increase in capillary permeability^2^, intravascular hypovolemia^3–5^ coexists with fluid overload in the interstitium, and is recognized as an independent risk factor of mortality^6–11^.

In the context of high vascular permeability, and as stated by the revised Starling equation, the main determinant for fluid transfer from the vessel lumen to the interstitium is the hydrostatic pressure difference between the intravascular and interstitial compartments (net filtration pressure)^12^. Until such time as these pressures become equal, plasma is filtered through the endothelium towards the interstitium, where fluid accumulates to form edema^13^. On the other hand, the large volume of fluid continuously leaking into the interstitium contributes to the persistent hypovolemia (with the need for repeated fluid replacement), hemodynamic instability, and associated organ failure that characterize septic shock. We hypothesized that the application of external pressure might increase interstitial pressure, immediately reducing the filtration pressure gradient and thereby the filtered volume. A similar approach for edema prevention has previously been proposed in acute respiratory distress syndrome (ARDS) patients with high alveolo-capillary permeability and edema reduction with positive airway pressure^14^. The present pilot study aimed to test the proof of concept of external corporeal compression to control fluid balance (FB), and evaluate the feasibility and tolerance of this technique.

## Results

**Figure 1:**
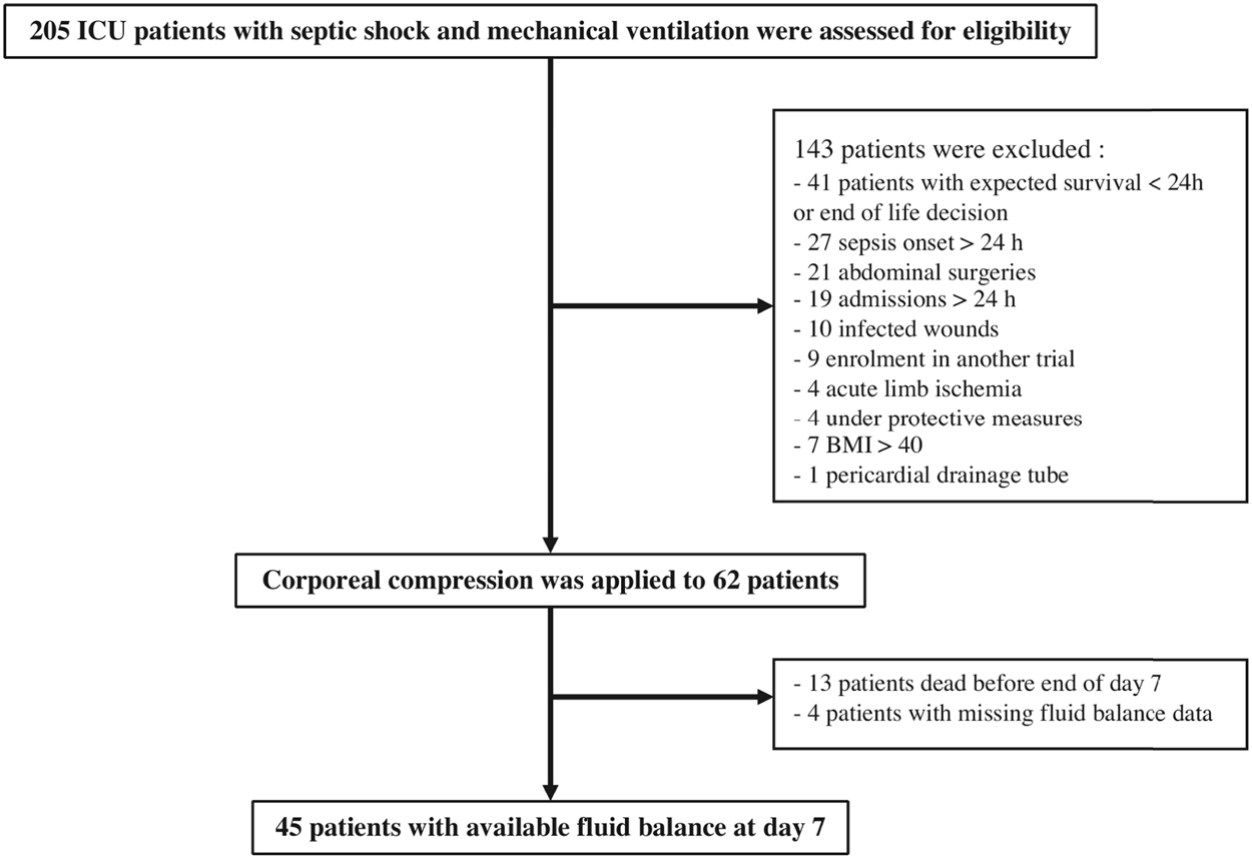
Flow chart of the study. BMI: body mass index; ICU: intensive care unit.

From February 2015 to December 2016, 62 patients were enrolled in the study. Thirteen patients (21%) died before day 7 and 4 patients (6.5%) were discharged from the ICU. Finally, 45 patients completed the protocol and had available fluid balance data at day 7 (Fig. 1). Table 1 shows the characteristics of the study population.

**Table 1:** Baseline characteristics of the study population.

Variable	N = 45
Age (years, median, IQR)	71 [63–74]
Women, n (%)	11 (24%)
BMI (kg/m^2^, median, IQR)	25 [22–31]
Comorbidities (n (%))	
Respiratory failure	7 (16%)
Heart failure	10 (22%)
End stage kidney disease	8 (18%)
SAPS II (median, IQR)	56 [49–66]
SOFA score (median, IQR)	12 [10–13]
Renal SOFA > 1 n (%)	24 (48%)
PaO2/FiO2 < 100 n (%)	12 (27%)
PaO2/FiO2 100–200 n (%)	24 (53%)
PaO2/FiO2 > 200 n (%)	9 (20%)
Sepsis origin, n (%)	
Pulmonary	34 (76%)
Urinary	8 (18%)
Biliary	2 (4%)
Unknown	1 (2%)
Culture positivity, n (%)	31 (68%)
Norepinephrine at inclusion, n (%)	44 (98%)
Time from admission to compression (hours, median, IQR)	8 [4–16]
Fluid balance at inclusion (median, IQR)	
Fluid intake (ml)	3050 [2000–4000]
Diuresis (ml)	448 [185–1100]
Fluid balance (ml)	2465 [1145–3140]

BMI, body mass index; SAPS II: Simplified Acute Physiology Score II; IQR: interquartile range; SOFA: Sequential Organ Failure Assessment.

### Bandage process

**Figure 2:**
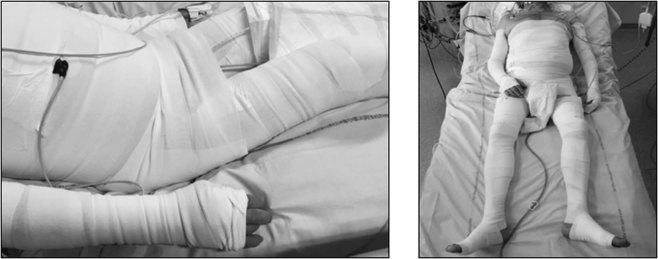
Example of a patient wearing corporeal compression. Photographs showing a patient wearing corporeal compression. Left side: bandages from the trunk overlap with the inferior limb bandages so that the hips and buttocks are covered. Patients consented for the possible open-access publication of anonymized images.

A median of three health care workers (from 2 to 6) were necessary to apply the bandages. The median time required to complete the bandage procedure was 20 minutes [17–25] using six 15cm-width and two 20cm-width bands. Bandages were handled for a median total time of 55 minutes per patient, mainly during the first day. An example of complete corporeal compression is shown in Fig. 2.

### Protocol adherence

The time between admission and the first bandage was 9 hours on average (Table 1). The median duration of the bandage was 4 days [3–7]. Compression was discontinued for the following reasons: achievement of the pre-defined target (22 patients; 42%); suspected or confirmed compression-induced discomfort (9 patients; 17%); ICU discharge (6 patients; 12%); death before day 7 (7 patients; 13%); presence of cutaneous ulcers (3 patients; 6%); required surgical procedure (5 patients; 10%); repetitive diarrhea (1 patient; 2%); end-of-life decision (1 patient; 2%).

### Primary outcome

**Figure 3:**
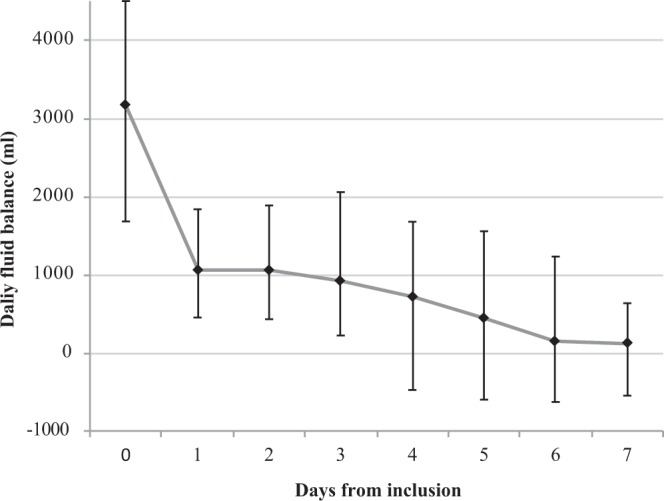
Daily fluid balance in the first 7 days after application of corporeal compression in 45 patients with available data. The daily fluid balance is showed for the first 7 days after application of corporeal compression in the 45 patients with data available throughout this period and included in the primary outcome analysis. N.B: day 0 balance includes the fluid balance reached before compression (in the ICU and/or emergency department).

The interim efficacy analysis was performed after the first stage of Simon’s plan (21 patients). The negative FB (<+500cc) rate at day 7 was observed in 13 of 21 patients (62%; 95% CI: 41,1%, 82,7%), allowing us to resume recruitment for the second stage as the threshold of 11 responses was reached. The final analysis was conducted as planned on the mITT population of 45 evaluable patients. A negative FB was observed in 29 of 45 patients with available data at day 7 (64%; 95% CI: 50.5%, 78.4%), exceeding the target of 26 responses. The day to day FB over the first 7 days is shown in Fig. 3.

### Secondary outcomes

All secondary outcomes (including feasibility and safety) were analyzed in the intention-to-treat population (n = 62). The cumulative FB at day 4 was 7280 ml [3300–9700], which included the pre-compression volume resuscitation (2495cc [1350–3500]). In the first week after inclusion, 49 patients (79%) had at least one day with FB < + 500 ml, occurring before day 4 in 34 patients (69%). Total SOFA score decreased to 8 [4–10] after 72 h versus 12 [10–13] at the time of inclusion. Respiratory and renal sub-scores also decreased at 72 hours (2 [1.5–3] versus 3 [3–4] and 1 [0–2.5] versus 2 [0–3], respectively). The median time to norepinephrine weaning was 2 days [1.5–3]. The median length of ICU stay was 11 days [4–22] with a median duration of mechanical ventilation of 6 days [4–13]. Overall mortality was 29% in-ICU, 34% at 28 days and 50% at 90 days.

### Historical control group

**Figure 4:**
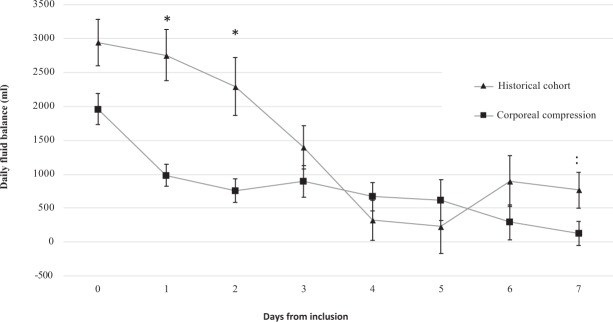
Daily fluid balance in the first 7 days after application of corporeal compression in 45 patients with available data versus historical cohort. Asterisks indicate a statistically significant difference between the two populations (p < 0.05). N.B: Fluid balance was recalculated in the corporeal compression patients to match the fluid balance calculation of historical controls (e.g. FB before ICU admission not included).

The fluid balance results of 50 patients from this study were compared to a group of 50 SOFA- and age-matched septic shock patients with acute kidney injury (AKI) from a historical cohort^15^. Demographic characteristics and fluid balance data for patients of both groups are summarized in Table 2. FB was significantly lower at 1, 2 and 7 days in the corporeal compression group (Fig. 4) This difference was not only the result of lower diuresis (which was expected, since all patients in the historical cohort had AKI) but was also due to significantly lower fluid requirements on days 1 and 2 (2330 vs. 2845, p < 0.001; and 2070 vs. 2835, p < 0.01, respectively), suggesting a role of compression in reducing capillary leak. Mortality was markedly lower in the corporeal compression group (18% vs 48% in the historical cohort at day 7, p = 0.001).

**Table 2:** Baseline characteristics and daily fluid balance compared between patients from a historical cohort of septic shock and corporeal compression-treated patients.

	Compression treatment	Historical cohort	p
**Baseline characteristics**	n = 50	n = 50	
Age (years, median, IQR)	71 [59–77]	71 [62–76]	0.90
Women, n (%)	12 (24)	13 (26)	0.82
SAPS II (median, IQR)	56.5 [49–72]	66 [55–80]	0.04
SOFA score (median, IQR)	12 [10–13]	12 [10–13]	0.72
**Fluid balance** (median, IQR)			
Inclusion day (≪day 0≫)	1785 [950–3000]; n = 50	2475 [1170–3780]; n = 45	0.07
Day 1	970 [300–1680]; n = 49	2405 [1355–3602]; n = 44	<0.001
Day 2	720 [200–1460]; n = 43	1919 [450–3380]; n = 38	0.002
Day 3	850 [50–2055]; n = 39	1437 [88–2088]; n = 36	0.33
Day 4	710 [−270–1320]; n = 37	253 [−1040–1540]; n = 28	0.31
Day 5	290 [−600–1545]; n = 37	13 [−855–1705]; n = 26	0.60
Day 6	157 [−650–1000]; n = 36	850 [−200–1850]; n = 23	0.09
Day 7	48 [−590–635]; n = 38	780 [120–1445]; n = 21	0.04

IQR: interquartile range; SAPS II: Simplified Acute Physiology Score II; SOFA: Sequential Organ Failure Assessment.

### Compression tolerance

Median interface pressure was 17 mmHg [14–20] (measured 34 times in 14 patients). Pressure-induced ulcers were observed in 16 patients (26%), with 12 at stage I (persistent redness) and 4 at stage II (dermabrasion). No stage III or IV lesions were observed. Most of the lesions (10 patients) were located on the tibial crest and/or knees, especially in patients treated with prone positioning. Compression had to be discontinued in only 3 patients due to cutaneous lesions.

The abdominal pressure level was 10.5 mmHg [8–13] before compression and did not change significantly 12–24 hours after bandage application (11 mmHg [8–14], p = 0.57). It remained stable during the following days (Supplementary Fig. 1). The plateau pressure did not change significantly after bandage application (20 [16–25] vs. 21 [17–25] mbar after 12 hours, p = 0.46), nor did thoraco-pulmonary compliance (Supplementary Fig. 2).

## Discussion

The present study is the first report of the use of corporeal compression to improve FB in septic shock patients. The primary endpoint, namely achieving a negative FB at day 7, was reached in 64% of the patients, suggesting that this treatment technique holds promise for future investigation in a phase III trial. At day 4, the cumulative FB was 7580 ml (including the FB recorded before compression), which is substantially lower than that reported by Boyd *et al*. at day 4 (11000 ml)^7^. This study, like ours, enrolled septic shock patients and recorded FB before patient inclusion. This discrepancy in the results could be due to a change in practice trending towards limiting fluid therapy. However, more recent studies with similar severity scores did not find significantly lower FBs^16^. Furthermore, when we compared FB in our cohort with a matched cohort of patients recruited more recently in our own centre, we confirmed the significantly lower FB in patients treated with compression, associated with a reduction in fluid requirements. The proportion of patients (69%) who had at least one day with FB < + 500 ml before day 4 also suggests a positive effect of compression. In an observational study, only 31% of septic shock patients had a negative FB before day 4, and they had a better survival rate^9^.

For this pilot study, we chose a single-arm design derived from cancer trials, the Simon’s plan. This type of design was developed specifically to optimize the sample size of early stage pharmaceuticals studies, for the preparation of phase III trials. At this stage, our study was not intended to provide a conclusive answer about the efficacy of the treatment. It confirmed the feasibility and safety of corporeal compression during sepsis, but its clinical benefits can only be assessed on intermediary endpoints compared to data from the literature and clinical experience.

The control of fluid overload is currently a major goal to improve sepsis management. The endothelial permeability is regulated by at least 5 known molecules during sepsis, with various signaling pathways^17^. Drugs targeting these regulatory mechanisms are currently under investigation^18^. However, it seems very difficult to target all these pathways involving various ligands and receptors and a mechanistic treatment such as this compression method might be the best approach for reduction of fluid overload and associated outcomes in sepsis.

This pilot study suffers from several limitations. First, although we used a historical cohort to serve as a reference, it does not constitute an adequate control group. The results of this study should be confirmed by an appropriate randomized controlled trial. Second, corporeal compression cannot reduce edema formation in vital organs such as the kidneys and lungs. However, when looking at the course of SOFA score in our population, our data shows that it tended to improve (especially the renal and respiratory sub-score) in the first few days when patients were wearing the compression. This suggests that organ edema does not increase during compression. Indeed, the purpose of compression is to prevent further leakage of intravascular fluid rather than filtrating excess interstitial fluid back into the circulation. On the other hand, we anticipated a risk of abdominal hypertension with compression, but abdominal pressure was not significantly increased and there seemed to be no consequence on the course of renal function.

Also, the outcome evaluation using FB at day 7 might bias the results, considering that the median duration of compression was 4 days in this study. Thus, 7 days might be too late for the outcome evaluation, with a risk of underestimating the efficacy of compression on FB. The choice of day 7 to evaluate the incidence of negative FB was empirical, in the absence of any published data on the duration of capillary leak in septic shock patients. Finally, the compression protocol was not optimal in terms of body surface coverage (especially in crucial zones where a large volume of edema accumulates, such as the buttocks and upper thighs), and in its effective duration (early discontinuation for various reasons). This could also lead to an underestimation of the potential efficacy of corporeal compression.

## Conclusion

This study demonstrates the feasibility of corporeal compression in septic shock and suggests a benefit on FB control, and might thus warrant further investigation in a phase III trial.

## Methods

### Patients

This prospective, multicenter, single-arm pilot study was approved by an independent ethics committee (Comité de Protection des Personnes Est-II) on 21^st^ January 2015. The study protocol was conducted in accordance with the relevant guidelines and regulations. It adheres to the Declaration of Helsinki and the guidelines for Good Clinical Practice. The study was registered with the European Union Drug Regulating Authorities Clinical Trials (EudraCT) and the French Health Products Safety Agency under the number 150017B-81. Each patient or their legal representative gave written informed consent after being provided with clear information.

Patients were recruited in 2 Intensive Care Units: one in Dijon (university teaching hospital), and one in Chalon-sur-Saone (non-academic hospital). The inclusion criteria were: age ≥ 18 years; ICU admission ≤24 hours; severe sepsis or septic shock (as defined by the Sepsis-2 definition^19^), ≤24 hours after the first hypotension; endotracheal intubation with mechanical ventilation. The exclusion criteria were: recent (≤2 weeks) abdominal/pelvic surgery, BMI > 40, local contraindication to bandages (i.e. acute limb ischemia, crush syndrome with rhabdomyolysis, limb compartment syndrome, infected leg ulcers), pregnant women, patients under protective measures, and moribund patients (expected survival ≤24 hours).

### External compression method

Non-elastic (“short stretch”) bandages were applied to cover 80% of the body surface. The type of bandage used (SOMOS Standard®; BSN medical, Germany; 15 cm or 20 cm width) exerts a low interface pressure (i.e. pressure measured between the skin and the fabric) with a low risk of skin lesions but good anti-edema efficacy^20,21^. The specifically trained paramedic team applied the bandages first on the four limbs. Then, on the abdomen and torso, the bandage was applied from the buttocks (overlapping the upper thigh bandage) up to a horizontal line delimited by the nipples (under the breasts in women).

### Study procedures

Compression was applied immediately after inclusion and bandages were changed once daily or more often if necessary. The compression was stopped when the FB was negative (difference between fluid intake and output ≤+500cc) for 2 consecutive days or at day 7, whichever came first. The fluid strategy was left at the discretion of the physician in charge of the patient, with no pre-defined protocol.

Skin tolerance of bandages was checked daily by visual skin inspection. The severity of ulcers was quoted using the National Pressure Ulcer Advisory Panel stages I to IV^22^. The presence of ulcers related to compression was considered a motive for discontinuation of bandage compression. Compression could also be stopped for any of the following reasons: intra-abdominal pressure (IAP; intravesical pressure checked twice daily^23^) increased to a level compatible with abdominal compartment syndrome (IAP > 15 mmHg + decrease in urine output)^24^; or airway pressure (plateau pressure) increased or dynamic compliance (in volume-controlled mode) decreased significantly with compression.

Fluid management was guided by the European Society of Intensive Care Medicine guidelines^25^. Repeated assessment of volume status (every 8 hours at least) was performed using a validated method such as inferior vena cava respiratory variations, pulse-pressure respiratory variations, and/or passive leg raising maneuver. Cristalloid fluid boluses were administered accordingly and repeated if necessary. The choice was given between a balanced solution (Ringer’s lactate) or saline.

### Primary and secondary outcomes

The primary outcome was the rate of patients with a negative FB at day 7 (regardless of the duration of compression).

The secondary outcomes were: cumulative FB during the first 4 days; death rate at 7, 28 and 90 days; course of SOFA score in the first 3 days; time to norepinephrine weaning; in-ICU length of stay; and the number of days under mechanical ventilation. The additional workload (daily handling time) for the technique was estimated (nurse-estimated time for bandage application or re-application). The reproducibility of interface pressure (between skin and bandage) was also verified, measured above the ankle (point B)^26^, when possible, by the investigator, with a specific device (Kikuhime®, Harada Co, Osaka, Japan).

### Design and statistical analysis

We used Simon’s two-stage design^27^ with a one-sided alpha error of 10% and a power of 90%. In a historical cohort of septic shock patients from our center^15^, 50% of patients had a negative FB at day 7; we used this reference to define the minimum response rate to achieve. We initially expected a response rate of 70% with compression. A sample size of 45 evaluable patients was needed under these assumptions^27^.

The primary outcome was planned for the modified intention-to-treat (mITT) population (i.e. all patients with FB data at day 7) and was reported with one-sided exact 95% confidence interval. The first interim analysis (first stage of Simon’s design) was conducted after inclusion of the first 21 evaluable patients. The trial could be terminated if 11 or fewer patients had a negative FB at day 7 (i.e. discontinuation for futility). If the threshold of 12 patients was met, the trial could continue with the enrollment of the next 24 patients to reach 45 evaluable patients. Overall, if a total of 26 responses or more were observed, the treatment would be considered a success^27^.

The secondary outcomes were planned for the intention-to-treat (ITT) population, including all enrolled patients. Categorical variables were expressed in percentage and continuous variables were expressed in median [interquartile range, IQR]. Survival was estimated by Kaplan-Meier method. Safety and tolerance are described for each adverse event. Statistical analyses were performed using SAS version 9.2 (SAS Institute Inc., Cary, NC, USA).

### Historical control group

In addition, to complete the study and validate our hypothesis with a view to planning a future randomized clinical trial, we used a group of historical controls from a cohort of septic shock patients from our center^15^ who were not managed by corporeal compression, to compare the pragmatic efficacy of the corporeal compression strategy on fluid balance. Patients from this cohort were matched for SOFA score (±2) and age (±5 years). The analysis was conducted on the whole population (i.e. not only patients with FB data through day 7) as daily FB was the focus of interest. FB calculation methods and means (time of origin, pre-ICU FB) had to be similar between the 2 groups and was thus recalculated for the study patients, when possible. Overall, the analysis was conducted on 50 patients from the study group and 50 matched control patients (Supplementary Fig. 3). Differences between the two groups were compared at each day using the Mann Whitney test for continuous variables and Fisher’s exact test for qualitative variables.

## Supplementary information

Supplementary figures

## Data Availability

The datasets generated and analyzed during the current study are available from the corresponding author on reasonable request.
