# Supplementary material for: Corporeal Compression at the Onset of Septic shock (COCOONs): a compression method to reduce fluid balance of septic shock patients
Source: Sci Rep. 2019 Aug 9;9:11566. doi: 10.1038/s41598-019-47939-2 (PMC6689006; doi:10.1038/s41598-019-47939-2)
Supplement: Supplementary file 1 — Supplementary figures [file 41598_2019_47939_MOESM1_ESM.pdf]

Corporeal Compression at the Onset of Septic shock (COCOONs): a compression method to reduce fluid balance of septic shock patients

Auguste Dargent[1,4], MD, Audrey Large[1], MD, Agnès Soudry-Faure, PharmD, PhD[2], Jean-Marc Doise[3], MD, Caroline Abdulmalak[3], MD, Lysiane Jonval[2], Pascal Andreu[1], MD, Jean-Baptiste Roudaut[1], MD, Sébastien Prin[1], MD, Pierre-Emmanuel Charles[1, 4], MD, PhD, Didier Payen[5], MD, PhD, Jean-Pierre Quenot[1,4,6], MD, PhD and the COCOONs study group

Supplementary figure 1: Course of intravesical pressure during compression

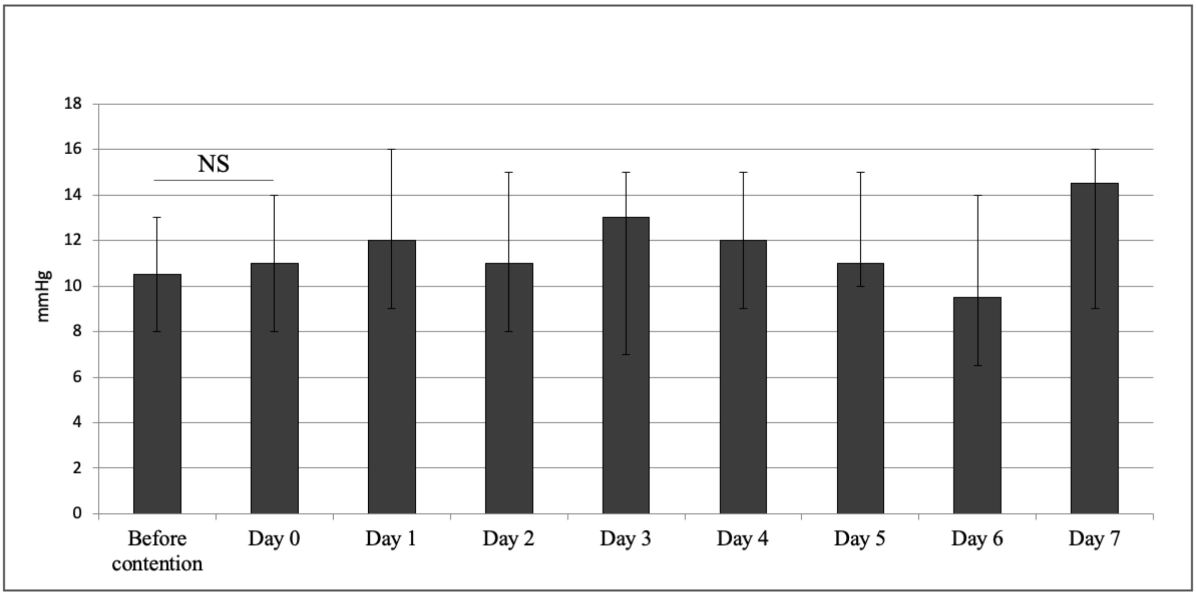

NS: non-significant difference between pressure in the first 24 hours after inclusion and pre-compression pressure

Supplementary figure 2: Course of plateau pressure and compliance during compression

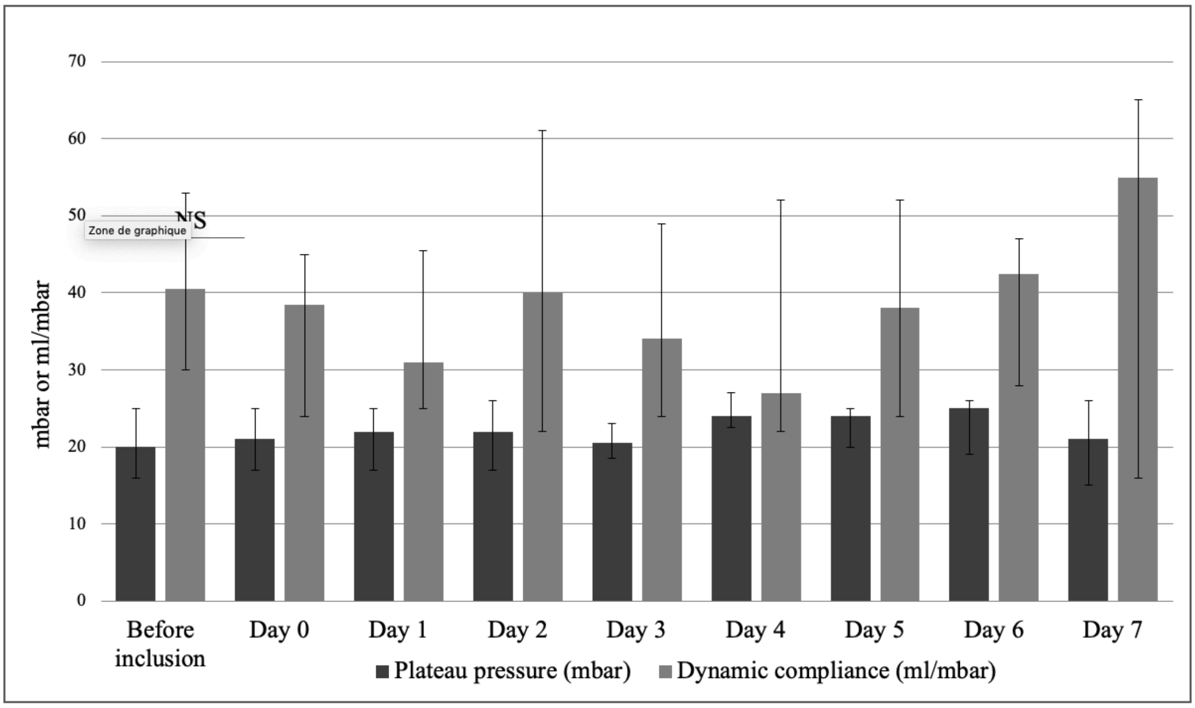

NS: non-significant difference between plateau pressure in the first 24 hours after inclusion and pre-compression pressure

Supplementary figure 3: Flow chart of the patients included in the complementary analysis with matching of study patients to an historical cohort on SOFA (±2 points) and age (± 5 years).

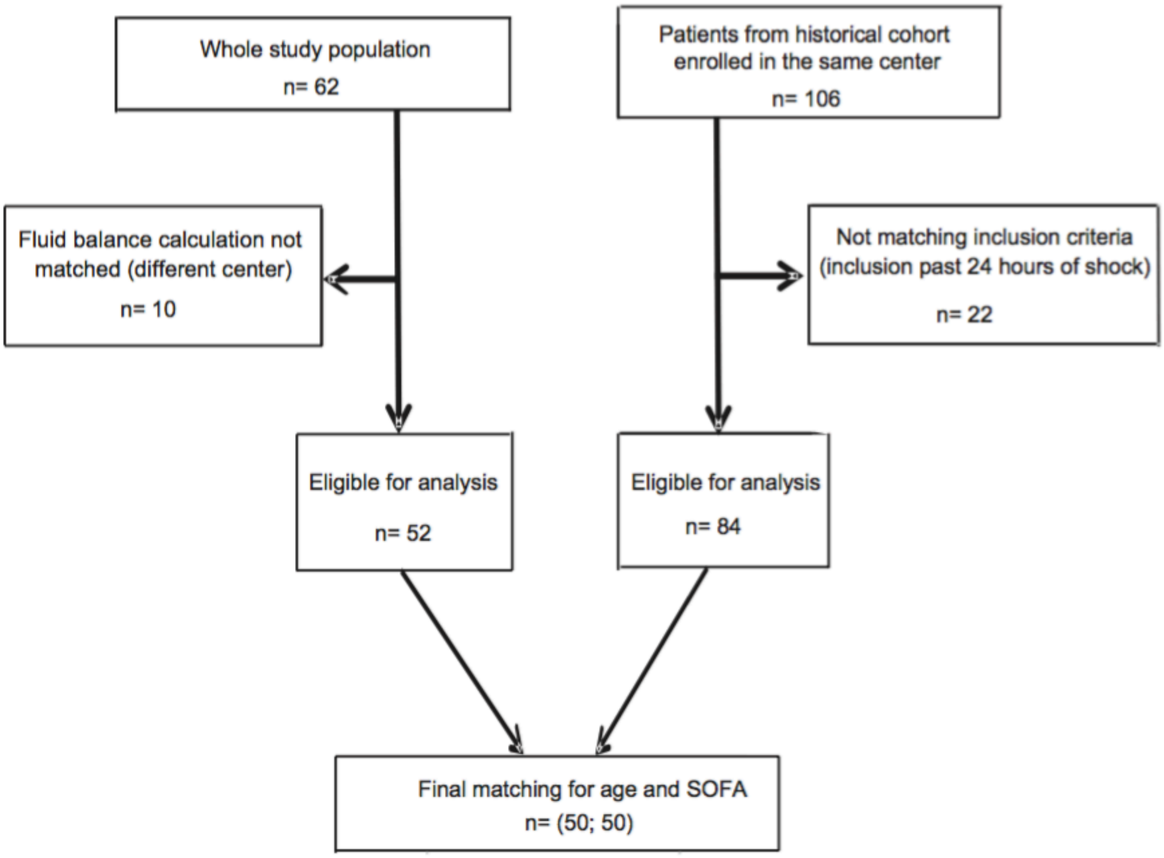

SOFA: Sequential organ failure assessment
